# Interstitial pneumonia microenvironment promotes metastasis to the mediastinal lymph nodes and lungs

**DOI:** 10.3389/fonc.2025.1657948

**Published:** 2025-10-14

**Authors:** Ryo Maeda, Mayu Inomata, Ryusei Yamada

**Affiliations:** Department of Thoracic and Breast Surgery, Faculty of Medicine, University of Miyazaki, Miyazaki, Japan

**Keywords:** lung cancer, interstitial lung disease, fibroblast, metastasis, pirfenidone

## Abstract

**Introductionhta:**

The prognosis of patients with lung cancer and interstitial lung disease (ILD) is worse than that of patients without ILDs; however, therapeutic options for ILD-associated lung cancer are severely limited. Although ILD is associated with an increased incidence of lung cancer, it is unclear whether the ILD lung environment affects the biological behavior of lung cancer.

**Methods:**

We tested our hypothesis that the lung environment of ILD is associated with the biological behavior and progression of lung cancer using an *in vivo* murine model of interstitial pneumonia (IP) and lung cancer.

**Results:**

The bleomycin-induced IP lung environment promoted metastasis to the mediastinal lymph nodes or contralateral lungs in an orthotopic model of lung cancer. The results of our *in vivo* experiments were supported by clinical data, which indicated that a significantly greater number of carcinomas with vascular invasion and lymphatic permeation, lymph node metastases, and intrapulmonary metastases were found in patients with clinical stage I non-small cell lung cancer and ILD than in those without ILD. In addition, pharmacological treatment of the IP lung environment with pirfenidone (PFD) inhibited tumor progression in the IP lung cancer model.

**Conclusion:**

We found that the IP lung environment promotes lung cancer metastasis. The results of this study may pave the way for further clinical studies on the use of PFD alone or in conjunction with conventional chemotherapy in patients with lung cancer and ILD.

## 1 Introduction

Interstitial lung disease (ILD) is detected in up to 13.5% of patients with lung cancer, with reported relative risks ranging from 7 to 14 (1–7). However, therapeutic options for lung cancer associated with ILD are very limited due to the potential for life-threatening acute exacerbation of ILD related to cancer treatments, such as radiotherapy, chemotherapy, and surgery (8–13). Therefore, the prognosis of patients with lung cancer and ILD is reportedly worse than that of lung cancer patients without ILD (14–18), and developing a new therapeutic strategy for patients with lung cancer and ILD is crucial.

In our previous clinical study (19), patients with combined pulmonary fibrosis and emphysema (CPFE) had more tumors with lymphatic permeation, vascular invasion, and lymph node metastases than those without CPFE; thus, we hypothesized that the lung environment of ILD itself was associated with an increased risk of lung cancer and influences the biological behavior and progression of lung cancer. In the present study, we aimed to identify a novel treatment strategy for patients with lung cancer and ILD using our *in vivo* murine model of interstitial pneumonia (IP) and lung cancer.

## 2 Materials and methods

### 2.1 Ethical considerations

The institutional review board approved data collection and analysis, and the requirement for written informed consent from each patient was waived. All animal experiments were performed in accordance with the protocols approved by the Institutional Animal Care and Use Committee. All efforts were made to minimize the number of animals used and their discomfort.

### 2.2 Patients

To confirm that patients with non-small cell lung cancer (NSCLC) and ILD had lymph node or lung metastases more frequently than those without ILD, we examined 516 consecutive patients clinically diagnosed with stage I NSCLC who underwent complete resection with systematic lymph node dissection between January 2011 and December 2020 at our institution.

### 2.3 Clinicopathological evaluations

Chest computed tomography (CT) was performed to detect ILD at the initial diagnosis, classify the stages of all patients, and measure the tumor size before surgery. ILD was considered present if there was subpleural or peribronchovascular reticulation characterized by traction bronchiectasis or bronchiolectasis with or without surrounding ground-glass opacities and honeycombing (20). Regional lymph node metastasis was clinically defined when the shorter diameter of a given lesion was ≥1.0 cm. Additional diagnostic testing, including brain magnetic resonance imaging, bone scintigraphy, and positron emission tomography combined with CT, was performed at the individual physician’s discretion according to the patient’s symptoms and clinical findings. Histological type was determined according to the World Health Organization classification (21). Disease stages were based on the tumor–node–metastasis classification of the International Union Against Cancer, eighth edition (22). Complete resection was defined as a gross and histological cancer-free surgical margin.

### 2.4 Murine model of interstitial pneumonia

Six-week-old female C57BL/6 mice were obtained from CLEA Japan (Tokyo, Japan). The mice were maintained in a pathogen-free facility. Female C57BL/6 mice aged 8 weeks were intraperitoneally anesthetized using pentobarbital sodium (Kyoritsu Seiyaku Co., Tokyo, Japan), followed by a single intratracheal injection of 3 mg/kg of bleomycin (BLM) sulfate (Wako, Osaka, Japan) in 50 μl of sterile phosphate-buffered saline (PBS), as described previously (23).

### 2.5 Cell culture

The mouse Lewis lung cancer (LLC) cell line was purchased from the Health Science Research Resources Bank (Osaka, Japan) and maintained in Roswell Park Memorial Institute 1640 medium supplemented with 10% fetal bovine serum (Thermo Fisher Scientific, Yokohama, Japan). Mouse pulmonary fibroblasts were extracted from C57BL/6 mouse lungs, as previously described (24).

### 2.6 Orthotopic LLC model

LLC-luciferase-expressing cells (4 × 10^4^) were suspended in PBS containing 15% Matrigel (BD Biosciences, San Jose, CA, USA) and injected into the parenchyma of the left lung lobe through the rib cage using a 29 G needle. To directly visualize the lung, a 4- to 5-mm incision was made in the skin under the left shoulder, which was then closed (25). Next, 7, 10, and 14 days after the injection of LLC cells, mice were intraperitoneally injected with 200 μl of d-luciferin (15 mg/ml, ViviGlo Luciferin; Promega, Madison, WI, USA) under anesthesia with 2% isoflurane. Bioluminescence images were then obtained using the *In Vivo* Imaging System(IVIS)Lumina II with Living Image ver. 3.2 (Xenogen, Alameda, CA, USA), following the manufacturer’s protocol.

### 2.7 Histological examination

Lung tissues were fixed in 4% formalin for 12–16 h, dehydrated, and embedded in paraffin. The sample blocks were sliced into 4-μm-thick sections and stained with hematoxylin and eosin (HE). The Masson’s trichrome (MT) assay was used to assess lung fibrosis. The Ashcroft score (26) was used to evaluate the degree of pulmonary fibrosis.

### 2.8 Immunohistochemistry

Lung tissue sections were incubated in blocking solution (10% goat serum in PBS) for 30 min at room temperature, followed by incubation with anti-mouse α-smooth muscle actin (α-SMA) – Cy3 (Sigma-Aldrich, St. Louis, MO, USA) monoclonal antibody.

### 2.9 Invasion assay

Matrigel invasion activity was assayed using the FluoroBlok Cancer Cell Invasion Assay System (BD Biosciences) in accordance with the manufacturer’s instructions. Briefly, tdTomato-labeled cells (5 × 10^4^) were seeded in serum-free culture medium onto Matrigel-coated filters with or without fibroblasts isolated from mouse lungs. Culture medium supplemented with 10% FBS was added to the lower chambers. After incubation in 5% CO_2_ at 37 °C for 16–20 h, the fluorescence intensity of the invaded cells was determined using a GENios microplate reader (Tecan, Männedorf, Switzerland).

### 2.10 Hydroxyproline assay

Collagen levels in the lung tissues were determined using the SIRCOL collagen assay (Biocolor Ltd., Carrickfergus, UK), according to the manufacturer’s instructions. Briefly, left lung lobes were homogenized, and collagen was solubilized in 0.5 M acetic acid. The extracts were incubated with Sirius red dye, and the absorbance was determined at 540 nm. The results were expressed as milligrams of collagen per left lung.

### 2.11 Pharmacological treatment

We examined whether pharmacological treatment with pirfenidone (PFD) blocks lung cancer progression promoted by the IP lung environment. PFD was the first anti-fibrotic agent approved for the treatment of idiopathic pulmonary fibrosis (IPF) in Japan. PFD (Shionogi & Co., Osaka, Japan) was suspended in a 0.5% carboxymethylcellulose (Nacalai Tesque, Kyoto, Japan) solution. PFD (300 mg/kg/day) was administered orally twice daily (7:00–10:00 and 19:00–22:00) and continued until dissection, as previously described (27, 28).

We intratracheally administered PBS or BLM with daily oral PFD. Two weeks after intratracheal administration, we injected LLC cells into the left lungs. Two weeks after the LLC cell injection, we evaluated the lungs of the four groups (PBS-vehicle, PBS-PFD, BLM-vehicle, and BLM-PFD).

### 2.12 Statistical analysis

All data are presented as mean ± standard deviation. Differences in categorical outcomes were evaluated using the χ^2^ test. Normally distributed continuous variables were compared using an unpaired *t*-test for two groups or one-way analysis of variance for multiple groups. All reported *P*-values were two-sided, and the significance level was *P* < 0.05.

## 3 Results

### 3.1 Correlation of ILD and clinicopathological characteristics in patients with clinical stage I NSCLC

Correlations between patients with ILD and postoperative pathological characteristics are shown in **Table 1**. Patients with ILD had a significantly greater number of moderately or poorly differentiated carcinomas and carcinomas with vascular invasion, lymphatic permeation, pleural invasion, lymph node metastases, and intrapulmonary metastases than those without ILD (**Table 1**).

**Table 1:** Correlation between interstitial lung disease (ILD) and the clinicopathological characteristics of patients with clinical stage I non-small cell lung cancer (n = 516).

Clinicopathological characteristics		ILD		P value†
		Absent	Present	
Total	516	449 (87)	67 (13)	
		Number of patients (%)		
Age (years)	< 70	232 (52)	30 (45)	0.292
	= 70	217 (48)	37 (55)	
Sex	Men	215 (48)	59 (88)	<0.001*
	Women	234 (52)	8 (12)	
Smoking history	Never-smoker	234 (52)	6 (9)	<0.001*
	Ever-smoker	215 (48)	61 (91)	
%VC	_=_ 80%	412 (92)	57 (85)	0.076
	< 80%	37 (8)	10 (15)	
Tumor laterality	Right	273 (61)	40 (60)	0.863
	Left	176 (39)	27 (40)	
Primary lobe	Upper or middle lobe	293 (65)	36 (54)	0.067
	Lower lobe	156 (35)	31 (46)	
Tumor size on chest CT (cm)	¾ 3.0 (T1)	361 (80)	50 (75)	0.273
	3.1- 4.0 (T2)	88 (20)	17 (25)	
KL-6	Value (mean ± SD)	248 ± 125	529 ± 438	<0.001*
	Not examined	48	1	
SUV_max_	Value (mean ± SD)	4.4 ± 4.6	8.8 ± 4.0	<0.001*
	Not performed	271	26	
Histological type	Adenocarcinoma	393 (88)	39 (58)	<0.001*
	Non-adenocarcinoma	56 (12)	28 (42)	
Histological differentiation	Well differentiated	160 (36)	9 (13)	<0.001*
	Moderately/poorly differentiated	289 (64)	58 (87)	
Lymphatic permeation	Absent	339 (76)	40 (60)	0.006*
	Present	110 (24)	27 (40)	
Vascular invasion	Absent	333 (74)	32 (48)	<0.001*
	Present	116 (26)	35 (52)	
Pleural invasion	Absent	333 (74)	40 (60)	<0.001*
	Present	116 (26)	27 (40)	
N status	N0	395 (88)	53 (79)	0.045*
	N1-3	54 (12)	14 (21)	
Intrapulmonary metastasis	Absent	443 (99)	60 (90)	<0.001*
Present	6 (1)	7 (10)		

ILD, interstitial lung disease; †chi-square test or Student’s t-test, numbers in parentheses are percentages, * indicates significance, VC, vital capacity; CT, computed tomography; KL-6, Krebs von den Lungen-6; SD, standard deviation; SUV, standard uptake value by positron emission tomography.

### 3.2 Influence of the IP lung environment on metastasis of cancer cell

We established a murine BLM-induced IP model. BLM was administered intratracheally to C57BL/6 mice. At 2 weeks after BLM administration, HE and MT staining of collagen revealed severe pulmonary damage and collagen deposition, which continued for at least 4 weeks after administration (**Supplementary Figure 1**).

We then established an orthotopic lung cancer model. We directly injected 4 × 10^4^ LLC cells into the left lungs of C57BL/6 mice. Fourteen days after implantation, the primary tumor was visible to the naked eye, and metastases to the mediastinal lymph nodes were observed (**Supplementary Figures 2A-C**). Although these mice lived 18–23 days after injection, no metastatic nodules were observed in distant organs, including the contralateral lung (**Supplementary Figure 2A**).

**Figure 1:**
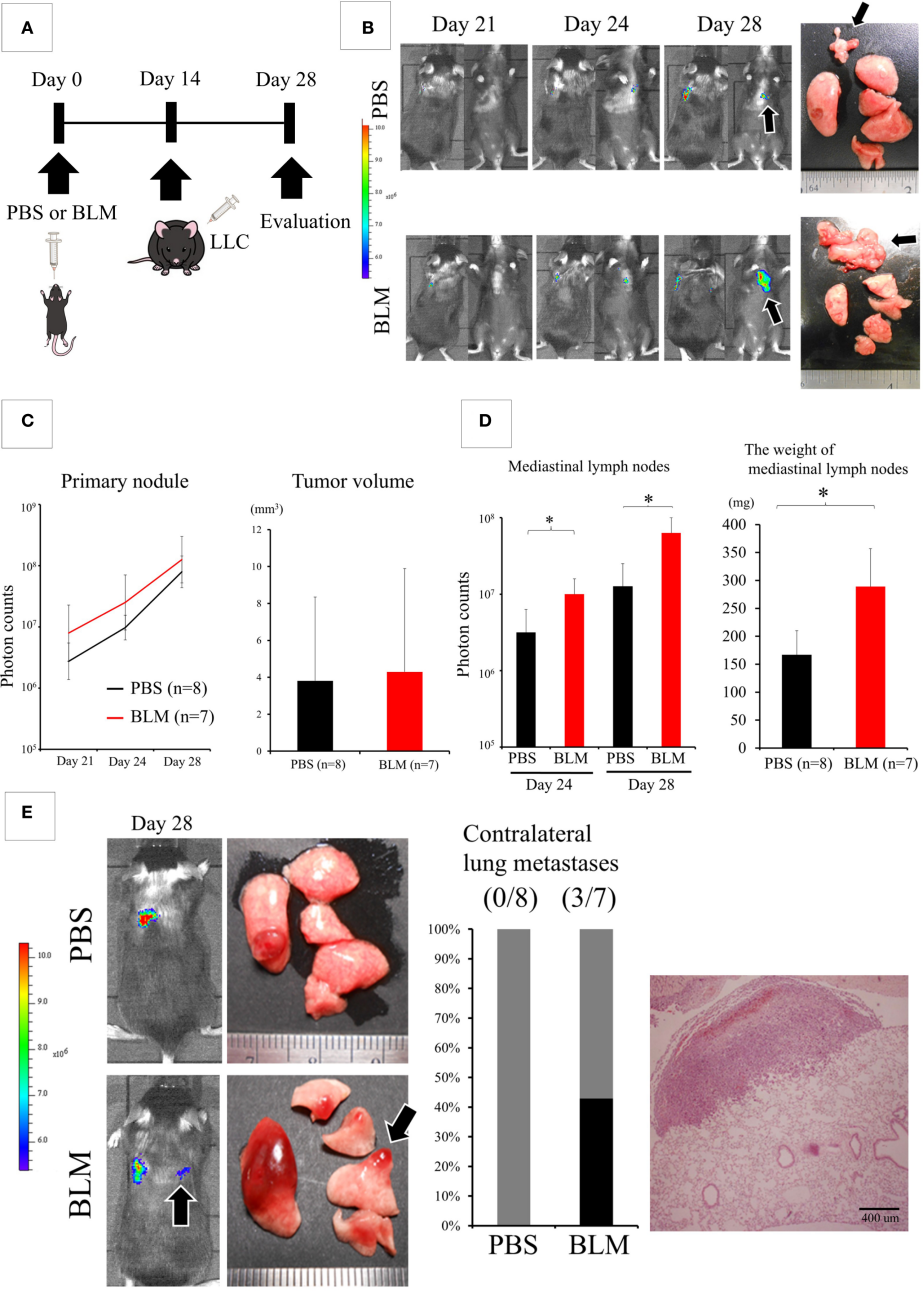
The lung environment of interstitial pneumonia (IP) promotes the metastasis of cancer cells. **(A)** Experimental design: Two weeks after the intratracheal administration of phosphate-buffered saline (PBS) or bleomycin (BLM), Lewis lung cancer (LLC) cells were directly injected into the left lung. Two weeks after LLC injection, the lungs were evaluated. **(B)** IVIS imaging of mice (left) and corresponding photos of the dissected lungs and mediastinal lymph nodes (right). Black arrows indicate metastatic foci in the mediastinal lymph nodes. Colored scale bar represents the intensity of bioluminescence (photon counts) from luciferase-expressing LLC cells. **(C)** Quantification of the primary tumor using bioluminescence (photon counts, left and volume, right). The differences in both fluorescent signals of the tumors on day 28 and the size of the primary tumor are not statistically significant (Student’s t-test). **(D)** Quantification of metastasis to mediastinal lymph nodes (photon counts, left or weight, right). *P < 0.05 (Student’s t-test). **(E)** IVIS imaging of mice (left) and corresponding photos of the dissected lungs (right) showing metastasis to the contralateral lungs (black arrows). The graph shows the frequency of mice with contralateral metastasis. Hematoxylin and eosin (HE) staining (left) shows a nodule of contralateral lung metastasis.

Using these models, we examined whether the BLM-induced IP lung environment affected the biological behavior of lung cancer. First, we intratracheally administered PBS or BLM. We injected LLC cells into the left lung 2 weeks later. The lungs of both groups were evaluated 2 weeks after LLC cell injection (**Figure 1A**). The difference in the primary tumor size did not significantly differ between the PBS and BLM groups; however, the weight of the mediastinal lymph nodes was significantly higher in the BLM group than in the PBS group (**Figures 1B-D**). Surprisingly, contralateral lung metastases were also observed in the BLM group (**Figure 1E**).

### 3.3 Effect of fibroblasts isolated from BLM-induced IP lung environment on the biological behavior of lung cancer cells

α-SMA-positive lung fibroblasts or myofibroblasts have been reported to play an important role in the pathogenesis of IPF (29). Indeed, α-SMA-positive cells were more frequently observed in the BLM-treated lungs than in control lungs (**Supplementary Figure 3A**). In addition, α-SMA-positive spindle-shaped cells were identified more often in the tumors of the BLM-induced IP model than in control PBS tumors (**Supplementary Figure 3B**). Therefore, we considered that α-SMA-positive fibroblasts or myofibroblasts are associated with lung cancer progression.

**Figure 2:**
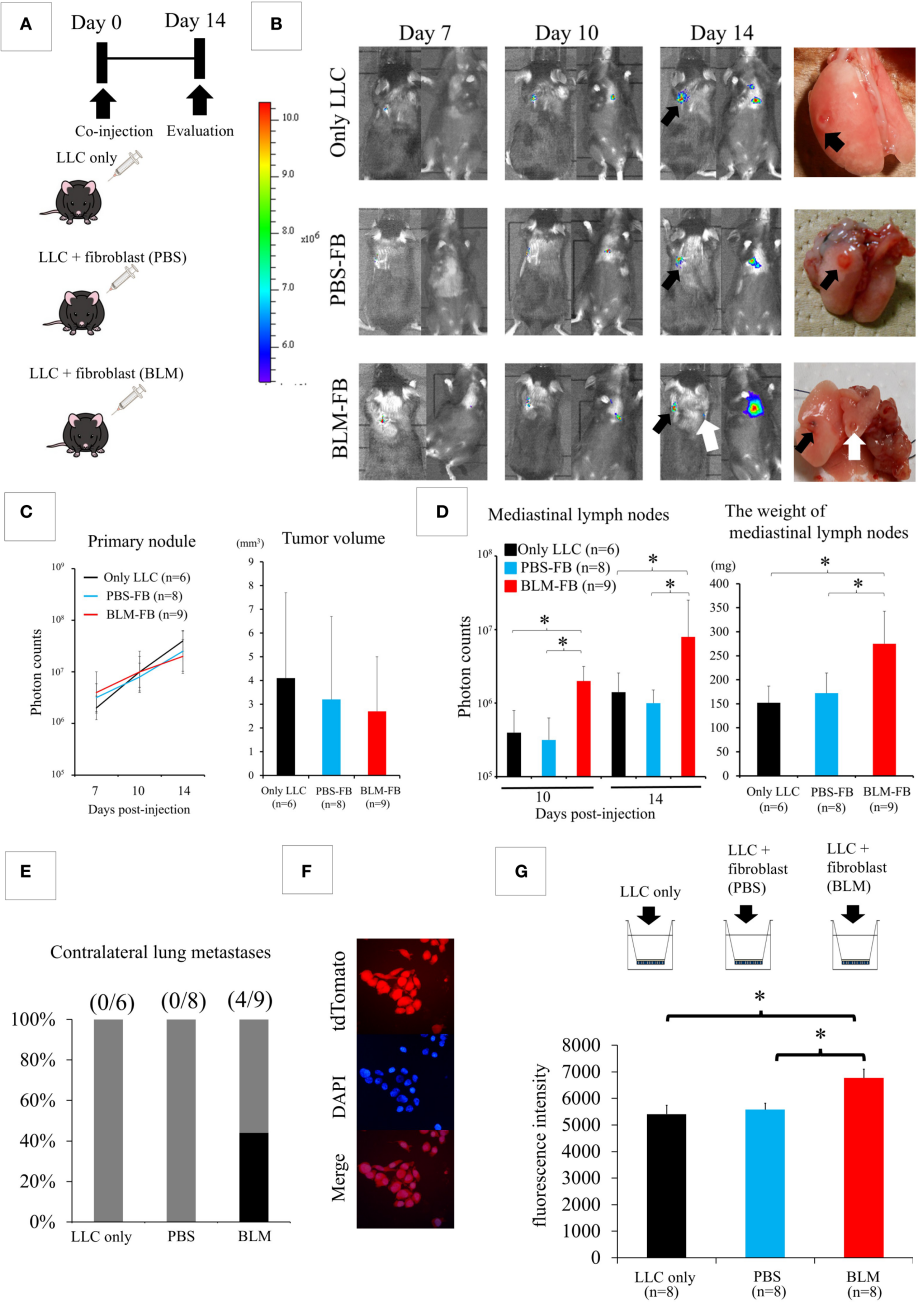
Fibroblasts isolated from bleomycin (BLM)-induced interstitial pneumonia (IP) affect the biological behavior of lung cancer cells. **(A)** Experimental design: Lewis lung cancer (LLC) cells are co-injected with medium alone, fibroblasts from the control phosphate-buffered saline (PBS) group, or fibroblasts from the BLM-induced IP group. **(B)** IVIS imaging of mice (left) and corresponding photos of the dissected lungs (right). Black arrows show the primary tumors in the left lung. The white arrow shows metastasis to the contralateral lung. **(C)** The differences in both fluorescent signals of the tumors on day 14 and the size of the primary tumor are not statistically significant among the three groups (one-way analysis of variance). **(D)** Quantification of metastasis to mediastinal lymph nodes (photon counts, left or weight, right). *P < 0.05 (Student’s t-test). **(E)** Contralateral lung metastases appear in the BLM-fibroblast group. **(F)** Fluorescent protein tdTomato is transfected into LLC cells **(G)** Matrigel invasion assay. *P < 0.05 (Student’s t-test).

Next, we isolated fibroblasts from both lungs 2 weeks after the intratracheal administration of PBS or BLM. Fibroblasts from BLM-induced IP lung environment showed a more spindle-shaped morphology and α-SMA-positive cells were more frequently detected than in control lungs (**Supplementary Figure 4**). As shown in **Figure 2A**, we directly co-injected 4 × 10^4^ LLC cells into the left lung of C57BL6 mice with medium alone, 4 × 10^4^ fibroblasts from the control PBS lung, and 4 × 10^4^ fibroblasts from the BLM-induced IP lung environment after dividing the mice into three groups (LLC-only, PBS-fibroblast, and BLM-fibroblast groups). Two weeks after the co-injection, the difference in the size of the primary tumor was not statistically significant among the three groups (**Figures 2B, C**); however, a significant increase in the weight of the mediastinal lymph nodes was observed in the BLM-fibroblast group (**Figure 2D**). In addition, contralateral lung metastases were observed only in the BLM-fibroblast group (**Figures 2B, E**). α-SMA-positive cells were more frequently detected in the tumors of the BLM-fibroblast group than in those of other groups (**Supplementary Figure 5**).

Since fibroblasts from BLM-induced IP lung environment promote lymph node metastasis of cancer cells, we examined whether fibroblasts affected the invasive capacity of LLC cells. After transfecting LLC cells with the fluorescent protein tdTomato (**Figure 2F**), we assessed the invasive capacity of cancer cells using a Matrigel invasion assay. LLC cells were seeded into the upper chamber after the addition of medium alone, in combination with fibroblasts from the PBS control lung, or in combination with fibroblasts from the BLM-induced IP lung environment (**Figure 2G**). A significantly increased number of invading LLC cells was detected in the BLM-fibroblast group compared with that in the other groups (**Figure 2G**).

### 3.4 Effects of the pharmacological treatment of IP on the inhibition of lung cancer progression

HE and MT staining revealed that severe pulmonary damage and BLM-induced collagen deposition were suppressed by the administration of PFD (300 mg/kg/day) (**Supplementary Figure 6A**). Furthermore, the BLM-induced elevation of pulmonary hydroxyproline (an indicator of collagen levels) was significantly suppressed (**Supplementary Figure 6B**). According to Ashcroft’s method, quantitative histology also showed that PFD significantly attenuated the score when administered to BLM-treated mice (**Supplementary Figure 6C**).

**Figure 3:**
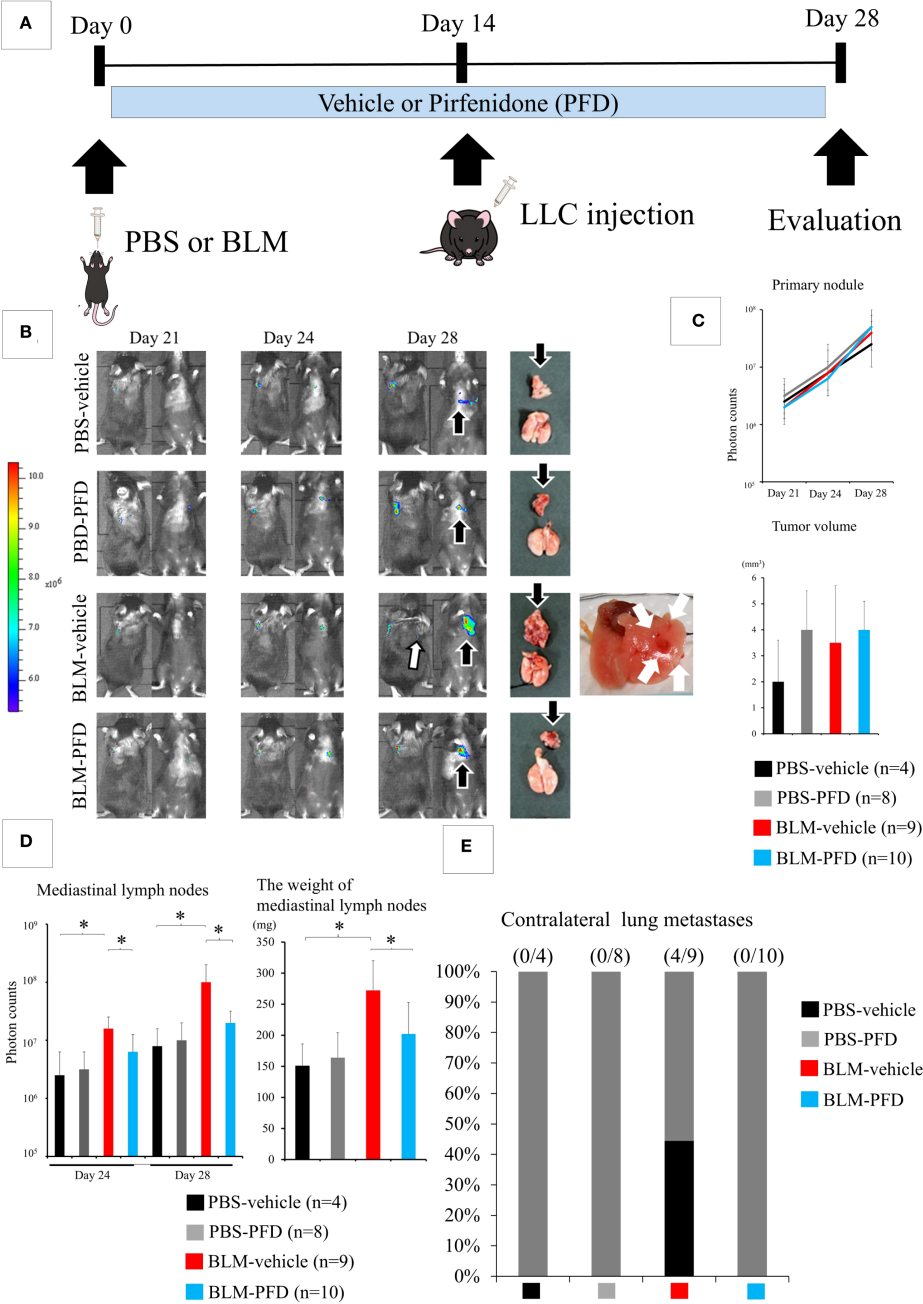
Pharmacological treatment of interstitial pneumonia (IP) leads to the inhibition of lung cancer progression. **(A)** Experimental design: Pirfenidone (PFD) is administered twice a day orally. **(B)** IVIS imaging of mice and corresponding photos of the dissected lungs and mediastinal lymph nodes. White arrows and black arrows show metastatic foci in the contralateral lungs and the mediastinal lymph nodes, respectively. **(C)** The differences in both fluorescent signals of the tumors on day 28 and the size of the primary tumor are not statistically significant among the four groups (one-way analysis of variance). **(C)** Significant decreases in the fluorescent signal and weight of the mediastinal lymph nodes are observed in the bleomycin (BLM)-PFD group compared with in the BLM-vehicle group. *P < 0.05 (Student’s t-test). **(D)** Contralateral lung metastases disappeared in the BLM-PFD group. PBS, phosphate-buffered saline. **(E)** Contralateral lung metastases disappeared in the BLM-PFD group.

The intratracheal administration of PBS or BLM with daily oral PFD, injection of LLC cells into the left lung 2 weeks after intratracheal administration, and evaluation of the lungs of the animals in the four groups (PBS-vehicle, PBS-PFD, BLM-vehicle, and BLM-PFD) 2 weeks after LLC cell injection is shown in **Figure 3A**. The difference in the size of the primary tumor was not statistically significant between the groups (**Figures 3B, C**); however, a significant decrease in the weight of mediastinal lymph nodes was observed in the BLM-PFD group compared to that in the BLM-vehicle group (**Figure 3D**). Contralateral lung metastases were only observed in the BLM-vehicle group (**Figure 3E**).

## 4 Discussion

Although several reports have indicated that ILD is associated with an increased incidence of lung cancer (1–5), it remains unclear whether the IP lung environment affects the biological behavior of lung cancer. After successful development of targeted therapy and immunotherapy, these innovative treatment options are rapidly being applied for patients with advanced-stage lung cancer (30–32). However, despite this progress, lung cancer with comorbid IP remains poorly understood. This is primarily because clinical trials exclude lung cancer patients with comorbid IP due to the risk of anticancer therapy triggering the acute exacerbation of symptoms. A more detailed understanding of the efficiency of the metastatic process of cancer cells, including the surrounding microenvironment, is necessary to contribute to the development of better therapies and improve the outcomes of lung cancer patients with ILD. In the present study, we found that the BLM-induced IP environment promoted metastasis to the mediastinal lymph nodes or contralateral lungs in an orthotopic mouse model of lung cancer. The results of this *in vivo* model demonstrate the possibility that the lung environment of IP is associated with increased cancer progression. Our clinical data, which indicated that a significantly greater number of carcinomas with vascular invasion and lymphatic permeation, pleural invasion, lymph node metastases, and intrapulmonary metastases were found in patients with clinical stage I NSCLC and ILD than in those without ILD, also supports our hypotheses. These results will likely have a tremendous impact on the treatment strategies for patients with both lung cancer and ILD.

Several researchers have recently reported successful results with limited surgical resection for clinical stage I NSCLC tumors (33). However, locoregional recurrence after limited resection is common, even in patients with a pathologically confirmed negative surgical margin (34). This is probably due to intratumoral vessel involvement, with tumor cells spreading into the surrounding parenchyma via the lymphatic flow (35). In the present study, tumors with ILDs were significantly correlated with histologically invasive characteristics. Our clinical data indicated the need to be careful in proposing limited surgery to ILD patients with clinical stage I NSCLC.

Pharmacological treatment of the IP lung environment using PFD inhibited tumor progression in our *in vivo* model. The Assessment of PFD to Confirm Efficacy and Safety in Idiopathic Pulmonary Fibrosis study group reported that PFD significantly reduced disease progression as reflected by lung function, exercise tolerance, and progression-free survival in a phase III trial in patients with IPF (36). The results of PFD experiments in this study may pave the way for further clinical studies on the use of PFD alone or in conjunction with conventional chemotherapy in patients with lung cancer and IPF.

Cancer tissues are composed of cancer cells and the surrounding stromal cells. Stromal cells can support cancer cells. Fibroblasts, which are the major components of cancer stroma, are called cancer-associated fibroblasts (CAFs). These cells modulate cancer metastasis through synthesis and remodeling of the extracellular matrix, the production of growth factors, and their influence on angiogenesis, tumor mechanics, drug access, and therapy responses (37).

The formation of fibrotic foci consisting of myofibroblasts in the lungs is a prominent pathological characteristic of IPF (27). The progression of fibrosis is associated with fibroblast accumulation and fibroblast-to-myofibroblast differentiation (24, 25). Myofibroblasts are the cells responsible for fibrogenesis in pathologic environments in the lung and are characterized by the upregulation of α-SMA and collagen (38, 39). In the present study, α-SMA-positive fibroblasts isolated from the BLM-induced IP lung promoted metastases to the mediastinal lymph nodes or contralateral lung, functioning similarly to CAFs.

Our study has some limitations. We did not determine how fibroblasts from BLM-induced IP increase the invasive abilities of cancer cells. One possible mechanism is the direct extracellular binding of fibroblasts to cancer cells, which promotes cancer progression. Another possible mechanism is a soluble factor secreted by fibroblasts in BLN-induced IP lungs. Further studies are required to clarify the mechanisms by which fibroblasts from IP lungs increase the invasive capacity of cancer cells.

## 5 Conclusion

We found that the IP lung environment promotes lung cancer metastasis, which is at least partially dependent on fibroblasts in the IP lungs. Further evaluation of the roles of these fibroblasts in cancer development may reveal the mechanism underlying the interactions of cancer cells and fibroblasts in the IP lung environment and how such an environment promotes tumor progression. In addition, our findings suggest that either PFD alone or PFD in conjunction with conventional chemotherapy may be a novel therapeutic strategy for patients with lung cancer and ILD.

## Data Availability

The original contributions presented in the study are included in the article/**Supplementary Material**. Further inquiries can be directed to the corresponding author.
