# Supplementary material for: Interstitial pneumonia microenvironment promotes metastasis to the mediastinal lymph nodes and lungs
Source: Front Oncol. 2025 Oct 14;15:1657948. doi: 10.3389/fonc.2025.1657948 (PMC12558802; doi:10.3389/fonc.2025.1657948)
Supplement: Supplementary file 1 [file DataSheet1.pdf]

Supplementary Figure 1. Establishment of a murine model of bleomycin (BLM)-induced interstitial pneumonia (IP). Hematoxylin and eosin (HE) staining and Masson trichrome (MT) staining of collagen revealed severe pulmonary damage and collagen deposition following BLM administration. PBS, phosphate-buffered saline.

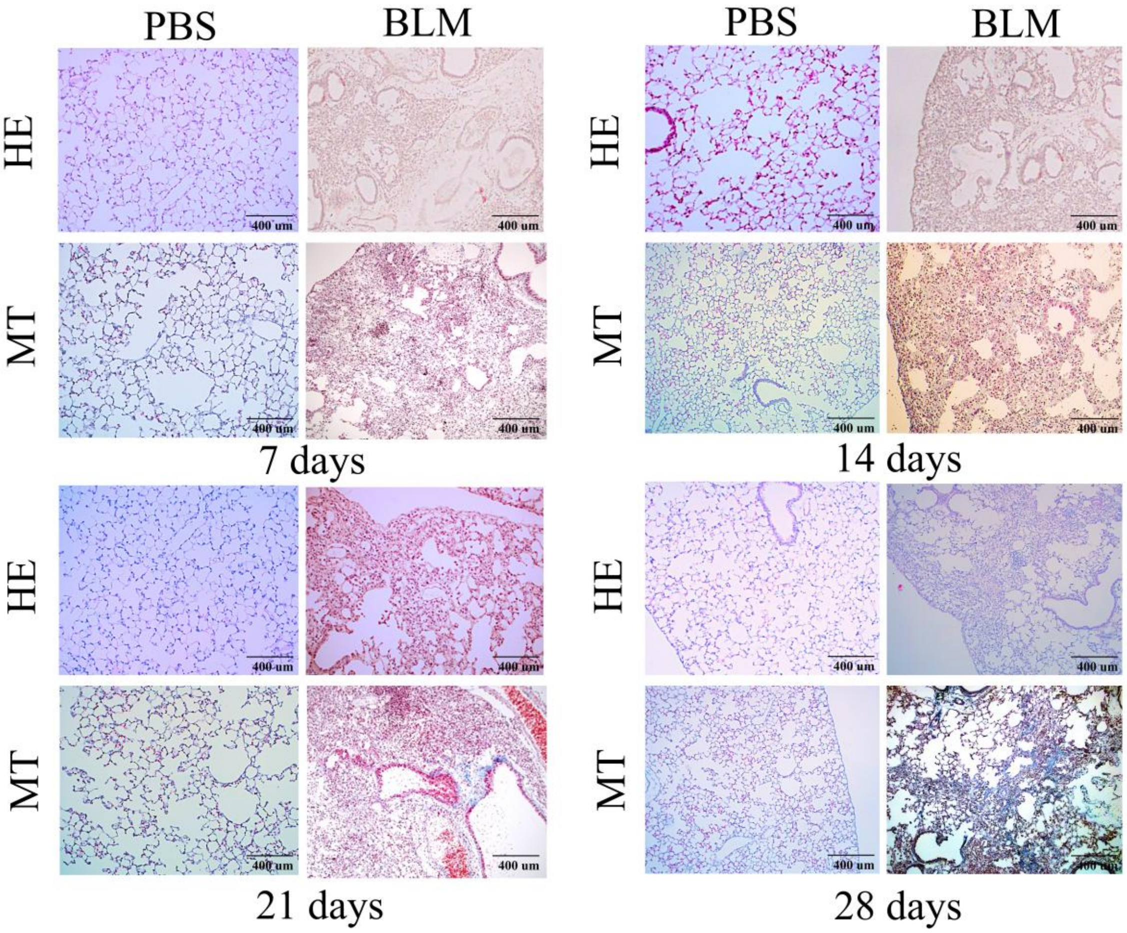
PBS
BLM
HE
MT
7 days
PBS
BLM
HE
MT
14 days
HE
MT
21 days
HE
MT
28 days
400 um

Supplementary Figure 2. (A) Establishment of the orthotopic model of lung cancer. IVIS imaging of mice (left) and photos of the dissected lungs (right). Colored scale bar represents the intensity of bioluminescence (photon counts) from luciferase-expressing Lewis lung cancer (LLC) cells. On day 18 after implantation, no metastatic nodules are observed in distant organs, including the contralateral lung. White arrows show the primary tumor. (B) White arrows show the primary tumor in the left lung on day 14 after implantation. (C) White arrows show metastases to the mediastinal lymph nodes on day 14 after implantation. Hematoxylin and eosin (HE) staining of the mediastinal lymph node on day 14 after implantation.

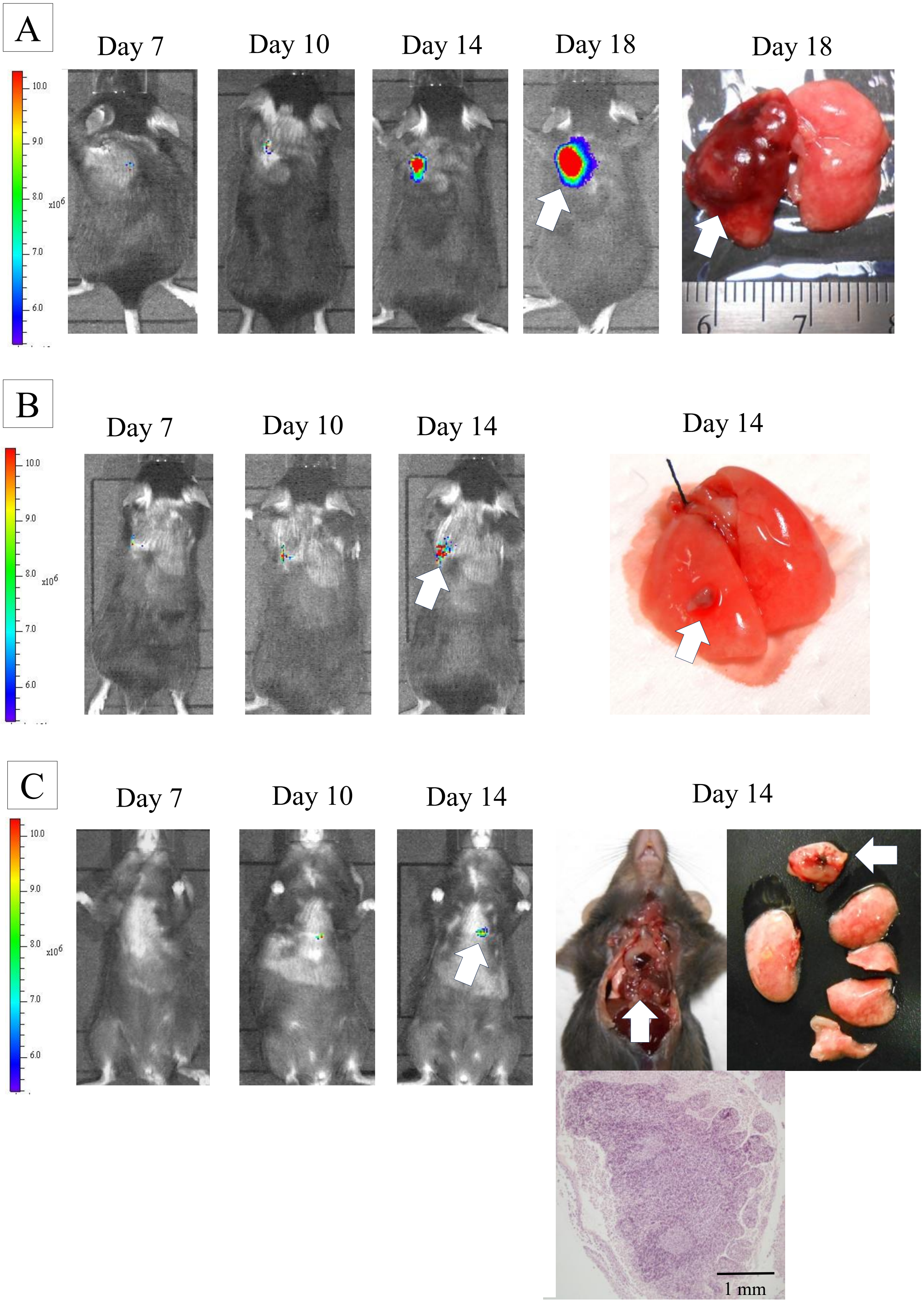
A
Day 7
Day 10
Day 14
Day 18
Day 18
10.0
9.0
8.0
x10^6
7.0
6.0
6
7
B
Day 7
Day 10
Day 14
Day 14
C
Day 7
Day 10
Day 14
Day 14
1 mm

1

Supplementary Figure 3. (A) α-Smooth muscle actin (α-SMA)-positive cells were more frequently observed in the lungs 14 days after the intratracheal administration of bleomycin (BLM) than in control lungs. **P* < 0.05 (Student's t-test). (B) α-SMA-positive spindle-shaped cells are detected more often in the tumors of the BLM-induced interstitial pneumonia (IP) model than control PBS tumors. **P* < 0.05 (Student's t-test).

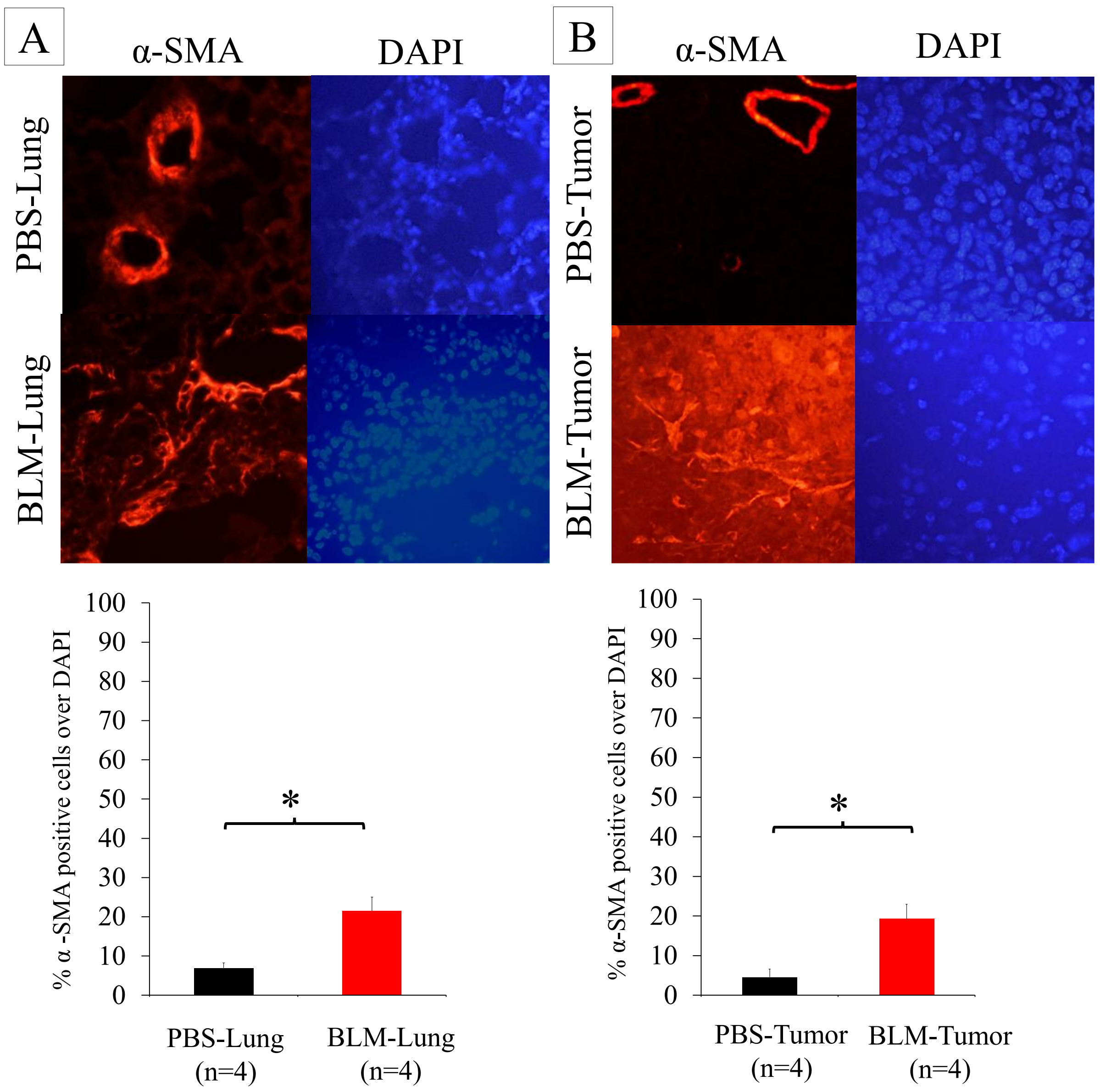
A
α-SMA
DAPI
PBS-Lung
BLM-Lung
B
α-SMA
DAPI
PBS-Tumor
BLM-Tumor
100
90
80
70
60
50
40
30
20
10
0
% α -SMA positive cells over DAPI
*
PBS-Lung (n=4)
BLM-Lung (n=4)
% α-SMA positive cells over DAPI
PBS-Tumor (n=4)
BLM-Tumor (n=4)

1

Supplementary Figure 4. Fibroblasts in the bleomycin (BLM)-induced interstitial pneumonia (IP) group show a more spindle-shaped morphology, and α-smooth muscle actin (α-SMA)-positive cells are more frequently detected than in controls using phosphate-buffered saline (PBS). $^*P < 0.05$ (Student's t-test).

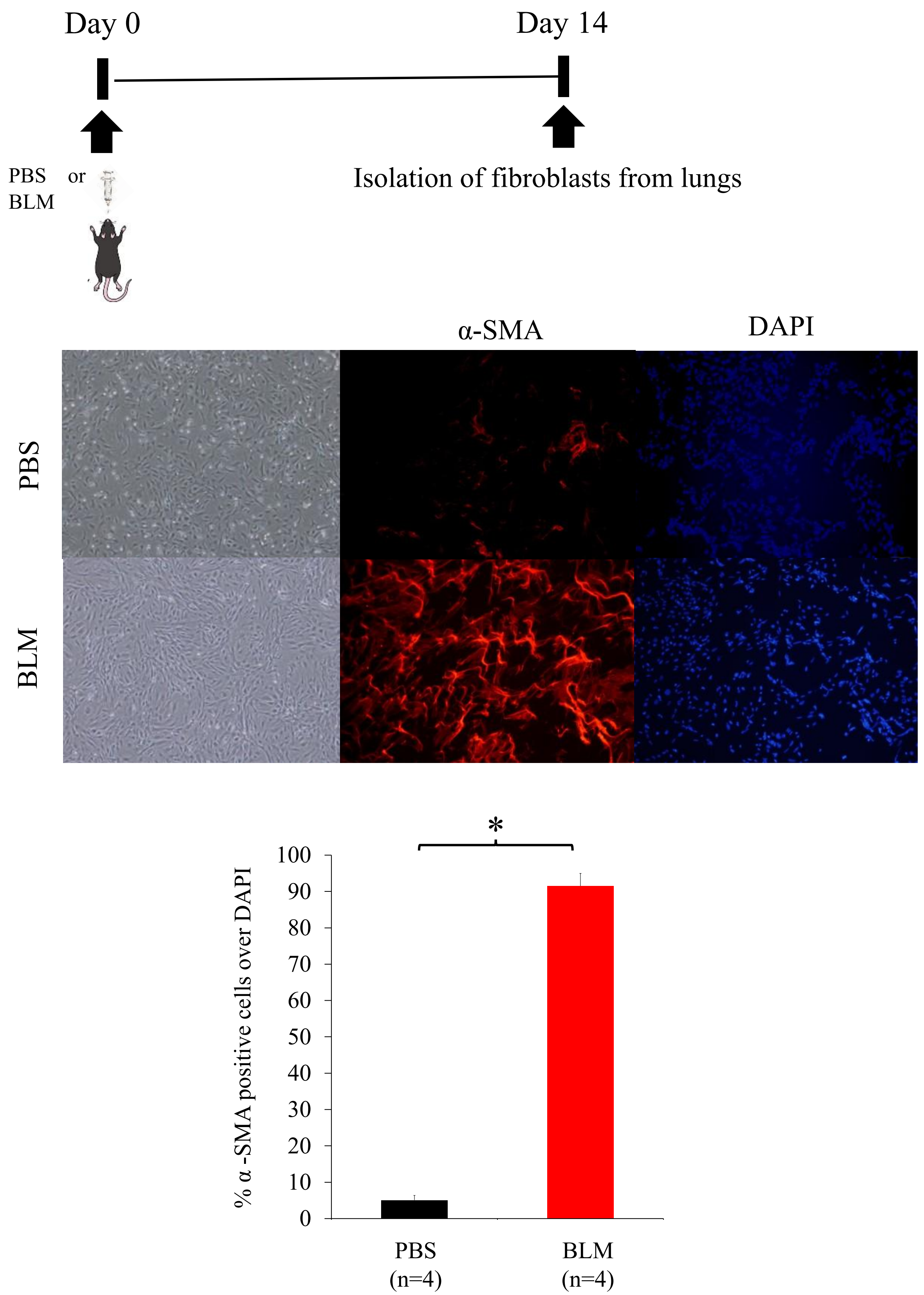
Day 0
Day 14
PBS or BLM
Isolation of fibroblasts from lungs
α-SMA
DAPI
PBS
BLM
*
% α -SMA positive cells over DAPI
0
10
20
30
40
50
60
70
80
90
100
PBS (n=4)
BLM (n=4)

Supplementary Figure 5. α-Smooth muscle actin (α-SMA)-positive cells are more frequently observed in the tumors of the bleomycin (BLM)-fibroblast (FB) group than in those of the other groups. $*P < 0.05$ (Student's t-test). HE, Hematoxylin and eosin; LLC, Lewis lung cancer.

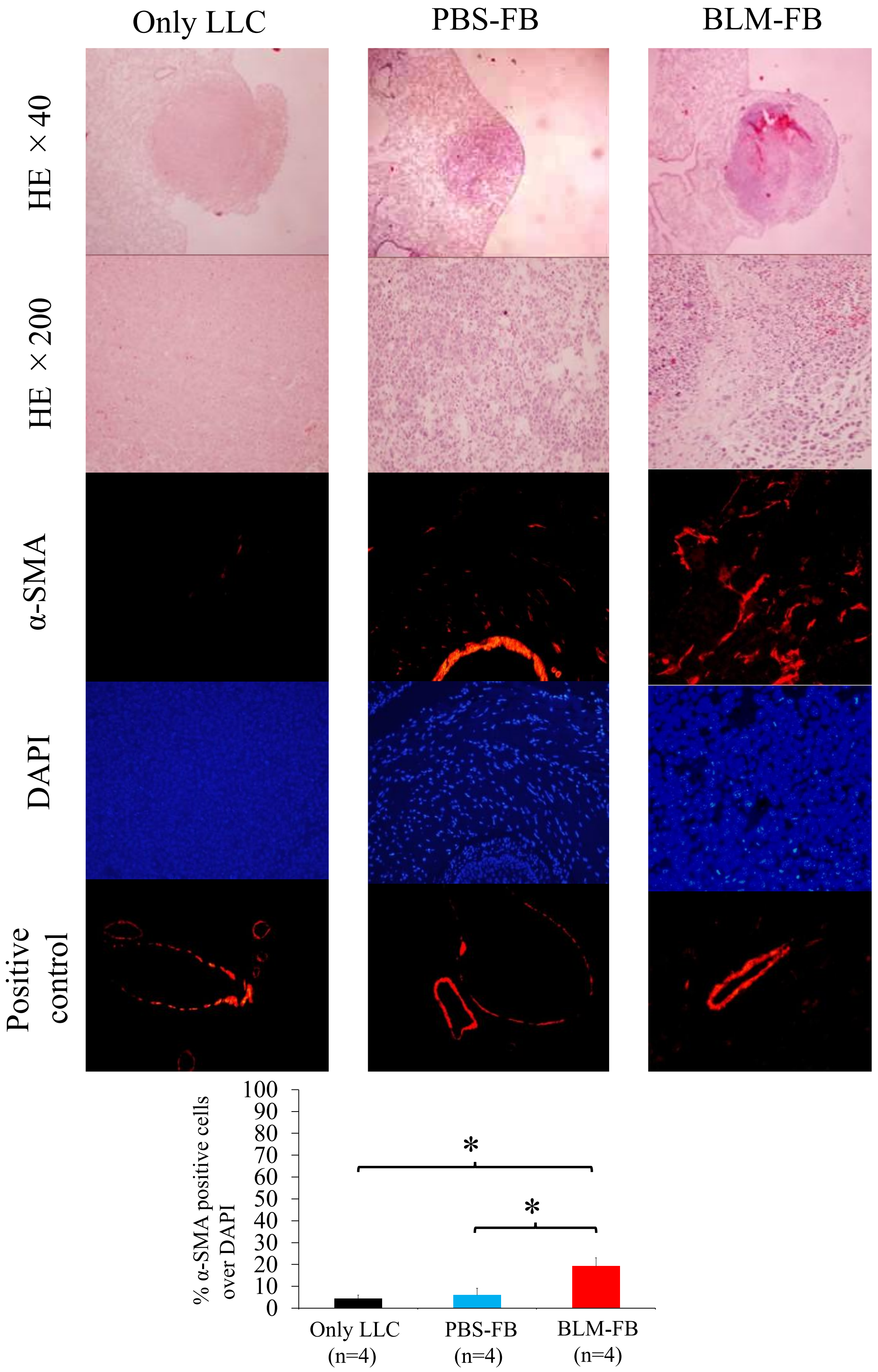
Only LLC
PBS-FB
BLM-FB
HE ×40
HE ×200
α-SMA
DAPI
Positive control
% α-SMA positive cells over DAPI
100
90
80
70
60
50
40
30
20
10
0
*
*
Only LLC (n=4)
PBS-FB (n=4)
BLM-FB (n=4)

1

Supplementary Figure 6. (A) Experimental design: Hematoxylin and eosin (HE) staining and Masson trichrome (MT) staining reveal that severe pulmonary damage and bleomycin (BLM)-induced collagen deposition are clearly suppressed by pirfenidone (PFD). (B) The BLM-induced elevation of pulmonary hydroxyproline is significantly suppressed. *P <0.05 (Student's t-test). (C) Quantitative histology according to Ashcroft's method shows that PFD significantly attenuates the score. *P <0.05 (Student's t-test). PBS, phosphate-buffered saline.

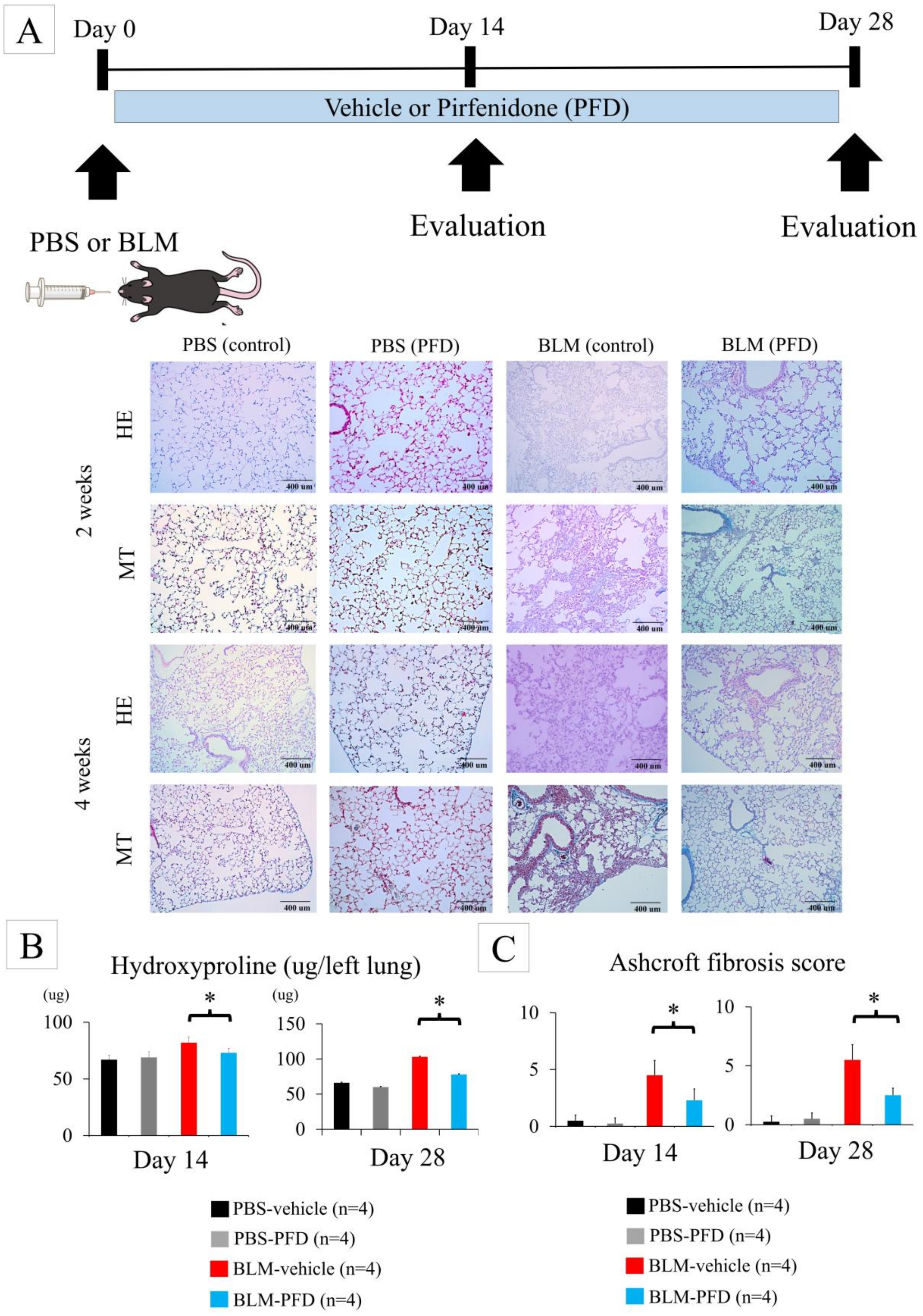
A
Day 0
Day 14
Day 28
Vehicle or Pirfenidone (PFD)
PBS or BLM
Evaluation
Evaluation
PBS (control)
PBS (PFD)
BLM (control)
BLM (PFD)
2 weeks
4 weeks
HE
MT
HE
MT
400 um
B
Hydroxyproline (ug/left lung)
(ug)
100
50
0
Day 14
(ug)
150
100
50
0
Day 28
*
PBS-vehicle (n=4)
PBS-PFD (n=4)
BLM-vehicle (n=4)
BLM-PFD (n=4)
C
Ashcroft fibrosis score
10
5
0
Day 14
Day 28
*
PBS-vehicle (n=4)
PBS-PFD (n=4)
BLM-vehicle (n=4)
BLM-PFD (n=4)
